# Supplementary material for: The Anti-Müllerian Hormone as Endocrine and Molecular Marker Associated with Reproductive Performance in Holstein Dairy Cows Exposed to Heat Stress
Source: Animals (Basel). 2024 Jan 9;14(2):213. doi: 10.3390/ani14020213 (PMC10812537; doi:10.3390/ani14020213)
Supplement: Supplementary file 1 [file animals-14-00213-s001.zip › animals-2745595-supplementary.pdf]

Supplementary Table S1. Summary of previous results about GWAS for serum and plasma AMH concentration including references, number of SNPs, chromosomal position, candidate genes and heritability.

Reference	No. SNPs	Chromosome (Position Mb)	Candidate Genes	Heritability ($h^2 \pm SE$)
Nawaz et al., 2018	11	BTA11 (92.059-99.413)	DENND1A, NR5A1, NR6A1, PTGS1, NDUFA8	0.36 ± 0.03
	1	BTA20 (25.689)	FST	
Gobikrushanth et al., 2018	44	BTA7 (22.498-23.120)	AMH	0.46 ± 0.31
	513	BTA11 (88.859-102.944)	FREM2	
	86	BTA20 (61.871-67.891)	FGF18, MTRR	
	1	BTA25 (97.149-97.360)	EEF2K	
Gobikrushanth et al., 2019	16	BTA7 (21-359-21.886)	AMH	0.45 ± 0.05
	52	BTA11 (92.051-101.918)	WDR38	
Grigoletto et al., 2020		BTA5 (97.149-97.360)	GPR19, CREBL2, DUSP16, BORCS5	0.28 ± 0.07
